# Enhanced Nesquehonite Formation and Stability in the Presence of Dissolved Silica

**DOI:** 10.1021/acs.est.3c06939

**Published:** 2023-12-27

**Authors:** Rasesh Pokharel, Iasmina C. Popa, Yannick de Kok, Helen E. King

**Affiliations:** † Department of Earth Sciences, Utrecht University, Princetonlaan 8a, 3584CB Utrecht, The Netherlands; ‡ Copernicus Institute of Sustainable Development, Utrecht University, Princetonlaan 8a, 3584CB Utrecht, The Netherlands

**Keywords:** attenuated total reflectance infrared spectroscopy, olivine carbonation, hydrated Mg-carbonate, carbon sequestration, transformation, precipitation, green cement, enhanced weathering

## Abstract

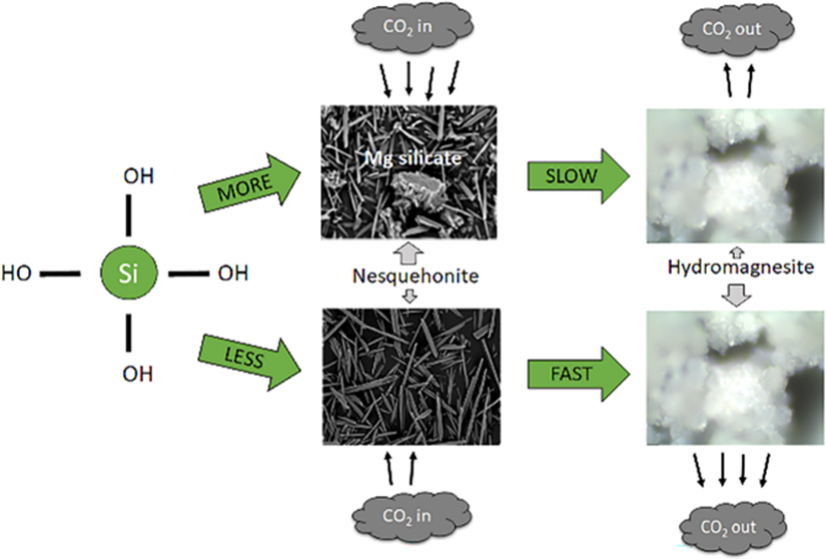

One possible carbon dioxide sequestration strategy is via the carbonation of dissolved Mg^2+^ obtained through olivine ((Mg,Fe)_2_SiO_4_) dissolution. However, silica is also produced during the breakdown of olivine. This component may have a detrimental effect on the yield of Mg-carbonate as Mg^2+^ incorporation into complex Mg silicate phases would limit CO_2_ uptake by this system. Yet this potential competition is currently not considered. Here, we use crystal growth experiments at temperatures applicable for potential coastal applications to test the effect of silica on the formation of the hydrated Mg-carbonate phase nesquehonite (MgCO_3_·3H_2_O). Solution chemistry analysis coupled with phase identification demonstrates that the presence of silica in the solution can actually assist the formation of nesquehonite and increase its yield by as much as 60 times. Our findings suggest that the presence of silica changes interfacial stabilities, lowering the energetic barrier for nesquehonite nucleation. In addition, in situ attenuated total reflectance-Fourier transform infrared spectroscopy (ATR-FTIR) transformation experiments demonstrated that nesquehonite precipitating in a solution containing a high concentration of dissolved silica exhibits enhanced stability against its transformation into hydromagnesite. These findings will help to better constrain what we expect for applications of olivine during carbon remediation strategies as well as assist yields for industrial applications that use Mg-based cement as building materials to facilitate a CO_2_-neutral or negative footprint.

## Abstract 2

This study shows the critical significance of dissolved silica (Si) in enhancing the precipitation and stability of nesquehonite, a hydrated magnesium carbonate mineral. This finding has implications for the process of CO_2_ sequestration via ex situ carbon mineralization, as well as for the production of magnesium-based green cement.

## 1 Introduction

Climate change currently sits at the forefront of global attention and as such significant efforts are being made to keep global warming at less than 1.5 °C above preindustrial levels.^1^ One method for storing CO_2_ that we generate or that is extracted from the atmosphere is via carbon mineralization, where CO_2_ or its dissolved species react with minerals to form solid and stable carbonate materials. The main advantage of this process is that the carbonate materials formed are end-products, thermodynamically stable over geological time periods (millions of years),^2^ and have alternative industrial uses such as green cement or concrete.^3,4^ Recent studies show promising results in using CO_2_ mineralization in the production of Mg-based cement in order to reduce the carbon footprint of the cement industry, which is responsible for 5–8% of global anthropogenic CO_2_ emissions.^5−7^ One potential process that is inspired by the natural weathering of mafic or ultramafic rocks is the reaction of the Mg-rich end member of the olivine mineral series.^8−12^ Here, the dissolution of olivine results in the local enrichment of Mg^2+^ with a concomitant increase in the solution pH, potentially facilitating the formation of Mg-bearing carbonate minerals through interaction with dissolved carbonate species. However, not only is Mg^2+^ released during the dissolution of olivine, but also silica. This produces a second possible Mg^2+^ sink as silica-rich Mg-bearing solids are also expected to form in such a system, e.g., sepiolite (Mg_4_Si_6_O_15_(OH)_2_·6H_2_O), talc (Mg_3_Si_4_O_10_(OH)_2_), or lizardite (Mg_3_(Si_2_O_5_)(OH)_4_), depending on the system temperature. If these phases form, carbonation yields would be reduced at temperatures relevant to the Earth’s surface up to high temperatures above 100 °C and low CO_2_ partial pressures.^11,13−15^ Thus, for the application of olivine to natural environments, this will be a significant effect that is currently underexplored in the literature.

At temperatures below 100 °C, anhydrous magnesite (MgCO_3_) formation is effected by kinetic inhibition, resulting in the preferential formation of metastable precursor phases such as nesquehonite (MgCO_3_·3H_2_O) and hydromagnesite (Mg_5_(CO_3_)_4_(OH)_2_·4H_2_O).^16,17^ Consequently, these initial hydrated phases are commonly observed in regions where ultramafic weathering occurs under Earth’s surface conditions.^17−20^ Nesquehonite is the most common Mg-carbonate mineral that can be precipitated from aqueous solutions at ambient pressure and temperature (i.e., 25 °C and moderate partial pressure of CO_2_).^16,21^ Although this phase is often overlooked, its potential in sequestering anthropogenic CO_2_ is being increasingly recognized.^20,22−24^ Thus, various projects are proposed that will use olivine for carbon sequestration under ambient conditions.^25−29^ However, the formation of metastable phases, such as nesquehonite, can slowly transform to other hydrated phases (such as hydromagnesite or dypingite) and ultimately to the most thermodynamically stable phase, magnesite, with time.^17,30,31^ The transformation of nesquehonite to more stable phases under ambient conditions involves the release of CO_2_ both in the air and underwater, which is not ideal for CO_2_ storage.^20,31,32^ Therefore, the lack of knowledge in the stability and precipitation of these hydrated Mg-carbonate phases represents a significant setback in the predictions we can make about the effectiveness of carbon sequestration.^33^

The main objectives of the research were to use controlled laboratory experiments to investigate the formation, stability, and phase transformation of nesquehonite in the presence of dissolved silica. We have specifically examined this process at high ionic strengths applicable for seawater settings, as there are several plans to use olivine in these settings as well as using high ionic strengths to minimize the hydration effects preventing Mg-carbonate formation. The experimental conditions were designed to mimic scenarios in which significant amounts of dissolved carbonates are already present, such as those resulting from the weathering of silicate minerals or existing in hyperalkaline natural environments. The overarching goal was to gain insights into how these conditions affect the precipitation and stability of magnesium carbonates in the presence of variable concentrations of dissolved silica under ambient conditions.

## 2 Methods

### 2.1 Solution Speciation Simulations

Prior to running the experiments, speciation calculations were performed to predict which mineral phases were likely to precipitate given the solution components and how the thermodynamic properties of the system may change across the experiment series. For this task, PHREEQC was used to estimate the saturation indices (SI) of the mineral phases in solution and the activity of the ions and potential ion pairs that may be present. Input solutions were the same as those used in the experiments. The Mg and carbonate concentrations, 0.08 M MgCl_2_ and 0.645 M NaHCO_3_, were slightly modified from those of Case et al.^21^ who successfully precipitated Mg-carbonate at similar concentrations under room temperature conditions. In our simulations, the temperature was set to 13 °C, a typical temperature for the surface of the North Sea,^34^ which is relevant to the industrial partners of the research project, and a concentration of 0.6 M NaCl was used to replicate seawater salinity. To obtain a pH that reflects that observed in the experiments (∼7.7), the dissolution of 0.645 M NaHCO_3_ (as nahcolite) was simulated. Five different solutions were simulated with varying silica concentrations: 0, 0.5, 1.5, 3, and 6 mM Na_2_SiO_3_. For all simulations, the Lawrence Livermore National Laboratory database (llnl.dat) was used.

### 2.2 Synthesis Experiments

Mg carbonates were synthesized in the laboratory using the double-decomposition method whereby a solution containing MgCl_2_·6H_2_O (Sigma-Aldrich, ACS grade) and NaCl (Sigma-Aldrich, ACS grade) was added to a solution of NaHCO_3_ (Sigma-Aldrich, ACS grade) with varying concentrations of Na_2_SiO_3_·9H_2_O (Sigma-Aldrich, >98%) to obtain the solutions described in Table 1. To do this, a stock solution of 250 mM Na_2_SiO_3_·9H_2_O was prepared and then subsequently diluted to create five different solutions with a volume of 300 mL and silica concentration of 0, 1, 2, 6, or 12 mM using double-deionized water in glass bottles. To each of these bottles, 21.67 g of NaHCO_3_ powder was added, resulting in a final concentration of 0.86 M carbonate in each bottle after being stirred with a magnetic stir bar until all of the solid had been dissolved. Finally, 100 mL of a 0.32 M MgCl_2_·6H_2_O with 2.4 M NaCl stock solution was added into each glass reactor such that the final reaction volume was 400 mL. This resulted in experimental solutions with 0.08 M MgCl_2_·6H_2_O, 0.645 M NaHCO_3_, and 0.6 M NaCl with either 0 (Cont-No-Si), 0.5 (Exp-0.5 mM-Si), 1.5 (Exp-1.5 mM-Si), 3 (Exp-3 mM-Si), or 6 (Exp-6 mM-Si) mM Na_2_SiO_3_·9H_2_O. While the silica concentrations used were significantly higher than the average concentration in the world ocean (around 70 μM),^35^ these conditions reflect scenarios where elevated silica levels can arise from the dissolution of quartz grains^36^ and olivine^37^ during enhanced silicate weathering experiments in coastal environments. The reactors were then sealed with rubber stoppers and Parafilm M to avoid interaction with atmospheric CO_2_ and placed onto a magnetic multiplate stirrer, set to 400 rpm, in a temperature-controlled room at 13 °C (to match the average temperature of the North Sea).^34^ Each solution composition was tested in duplicate. Each experiment was sampled at several time points during the experiments, including immediately after the solutions were combined. To sample, the glass bottle was removed from the stirring plate and shaken several times, and then 4 mL of the solution was extracted using a syringe and needle. Half of this sample was used to test the solution pH (using a Mettler Toledo SevenCompace pH meter with an accuracy of 0.002 pH units). The rest of the sample was filtered through a 0.2 μm pore size filter, directly acidified with 100 μL of concentrated HNO_3_ acid (Sigma-Aldrich, Suprapur), and then stored in a refrigerator at 5 °C until the end of the experiment. After the experiment was completed, these samples were diluted 10 times with quartz-distilled 0.7 M HNO_3_ acid using a Hamilton Microlab 600 semiautomated dilution system. Subsequently, the concentrations of Mg and Si were measured using PerkinElmer Avio 500 inductively coupled plasma optical emission spectroscopy (ICP-OES) at the Geolab in Utrecht University. The standards used for calibration were prepared with ICP multielement solution in artificial seawater to match the sample matrix. The analytical precision for both Mg and Si was better than 2%. Each solution was measured in duplicate.

**Table 1:** Summary of Solution Saturations from Speciation Modeling for Each Precipitation Experiment and Observational Results

		saturation indices^b^							
experiment^a^	Na_2_SiO_3_·9H_2_O (mM)	sepiolite	nesquehonite	lansfordite	hydromagnesite	magnesite	duplicate	precipitate first seen (day)^c^	extracted precipitate weight (mg)^d^
Cont-no-Si	0	na^e^	–0.62	0.18	0.91	2.51	A	none before termination at 18 days	
							B	15	5
Exp-0.5 mM-Si	0.5	2.52	–0.62	0.18	0.92	2.51	A	none before termination at 18 days	
							B	30	30
Exp-1.5 mM-Si	1.5	5.87	–0.55	0.25	1.33	2.58	A	23	113
							B	4	138
Exp-3 mM-Si	3	8.15	–0.48	0.31	1.73	2.65	A	2	270
							B	1	218
Exp-6 mM-Si	6	10.75	–0.37	0.42	2.43	2.76	A	2	389
							B	3	385

a Experiment ID of the precipitation experiments. All experiments contained 0.08 M MgCl_2_·6H_2_O, 0.645 M NaHCO_3_, and 0.6 M NaCl, but with variable Na_2_SiO_3_·9H_2_O concentrations (ranging from 0 to 6 mM).

b Saturation indices of various Mg silicate and carbonate minerals at the end of the experiment based on PHREEQC simulations.

c Time point at which precipitate was first observed.

d Weight of precipitate collected at the end of the experiment.

e na is not applicable.

The solids were harvested at the end of the experiment by centrifugation at 3200 rpm for 30 min, followed by washing twice with Milli-Q water, and were then centrifuged again. When no visible precipitate was observed, the solutions at the end of the experiment were put through a vacuum filtration setup by using a membrane with a pore size of 0.2 μm. The samples were then dried over 2 days in an oven at 30 °C and weighed. Phase identification was completed using a Bruker-AXS D8 ADVANCE X-ray diffractometer DAVINCI design with a LYNXEYE XE-T detector (with 192 measuring points) and Θ/Θ goniometer. The accuracy for this instrument was 0.01° 2Θ. In addition, Raman spectroscopy was used to identify the solids formed when their volume was insufficient for the X-ray diffraction (XRD) analysis. Here, a WITec Alpha 300 equipped with a Nd:YAG laser with a wavelength of 532 nm was used with a grating of 1800 grooves/mm. A spectral stitch with multiple windows between 150 and 1650 cm^–1^ was performed to obtain a good spectral resolution of the main lattice and internal modes relevant for carbonate minerals. Ten of these spectra were obtained, with a counting time of 5 s. They were then averaged to obtain a good signal-to-noise ratio for further analysis. This was followed by a single spectral window centered to capture the OH stretching spectral region (3000–3700 cm^–1^). Baseline removal and correction of faulty pixels was completed in the WITec Control Plus program version 5.3. Yields from the experiments were calculated using weight loss measurements from thermogravimetric analysis (TGA). A TGA701 LECO Thermogravimetric Analyzer, with a precision of 0.02% relative standard deviation, was used with a heat ramp rate of 1 °C/min up to 1000 °C in air. For consistency, each analyzed sample had the same initial weight of 0.1 g.

Scanning electron microscopy/energy dispersive X-ray spectroscopy (SEM/EDS) was used for both the morphological and elemental analysis of the solid samples. Prior to the analysis, all samples were mounted on a SEM sample holder using double-sided carbon tape. The samples were then coated with platinum (5 nm coating thickness) by sputtering with a plasma multicoater (Cressington 208HR Sputter Coater). All samples were examined with a Zeiss EVO 15 SEM, using a LaB6 (Lanthanum hexaboride) emitter and a Bruker Xflash 6/60 dual EDS detector. Acquisitions conditions were as follows: 15 kV accelerating voltage, 9.72–9.93 mm working distance, beam current 1 nA, and 10 to 1500× magnifications. For data analysis, the Espirit 2.3 software was used.

### 2.3 Transformation Experiments

Transformation of the precipitates upon heating was studied in situ using a Thermo Scientific Nicolet 6700 FTIR spectrometer equipped with a Pike GladiATR attenuated total reflectance (ATR) accessory at 25, 60, 70, 80, and 90 °C. The experiments were conducted in air or double-deionized water by using a fluid cell. Background spectra of the attenuated total reflectance-Fourier transform infrared spectroscopy (ATR-FTIR) system were collected prior to running the experiments. The solid sample was placed onto the diamond window and then either pressed to the window using a swivel press or, for experiments conducted in fluids, covered by the fluid cell into which ∼40 μL of deionized water was injected using a syringe. Air was allowed to escape from the opposite side of the fluid cell to the fluid injection until water could be seen emerging, at which point the cell was sealed by using Luer lock caps. The FTIR signal was obtained by averaging 32 scans at a resolution of 2 cm^–1^ that were acquired at regular intervals during the transformation experiment. Measurements of the water blank were also recorded at the temperatures of the experiments for later data interpretation (Figure S1). After the experiments were completed, the sample was extracted and the crystal was wiped clean using 90% isopropanol. The fluid cell was opened and rinsed with deionized water before being left to dry. SpectraGryph 1.2.16 Optical Spectroscopy Software was used to analyze the FTIR spectra removing the baseline. Subtraction of the water spectrum was completed where necessary for further data analysis using Omnics software supplied by Thermo Fisher.

When a phase transformation was detected, the 1099 and 1113 cm^–1^ bands in the FTIR spectra were fitted using a linear baseline correction on the 1150–1050 cm^–1^ spectrum region. The intensity of the 1099 cm^–1^ band was then divided by the intensity of the 1113 cm^–1^ band to obtain their ratio. The negative of a natural logarithm^38^ was applied to that ratio to generate the rate of change. These rates were then used in an Arrhenius plot to obtain the activation energies associated with the transformation of the crystallization products grown in the presence of different concentrations of dissolved silica.

## 3 Results and Discussion

### 3.1 Precipitation Experiments

**Figure 1:**
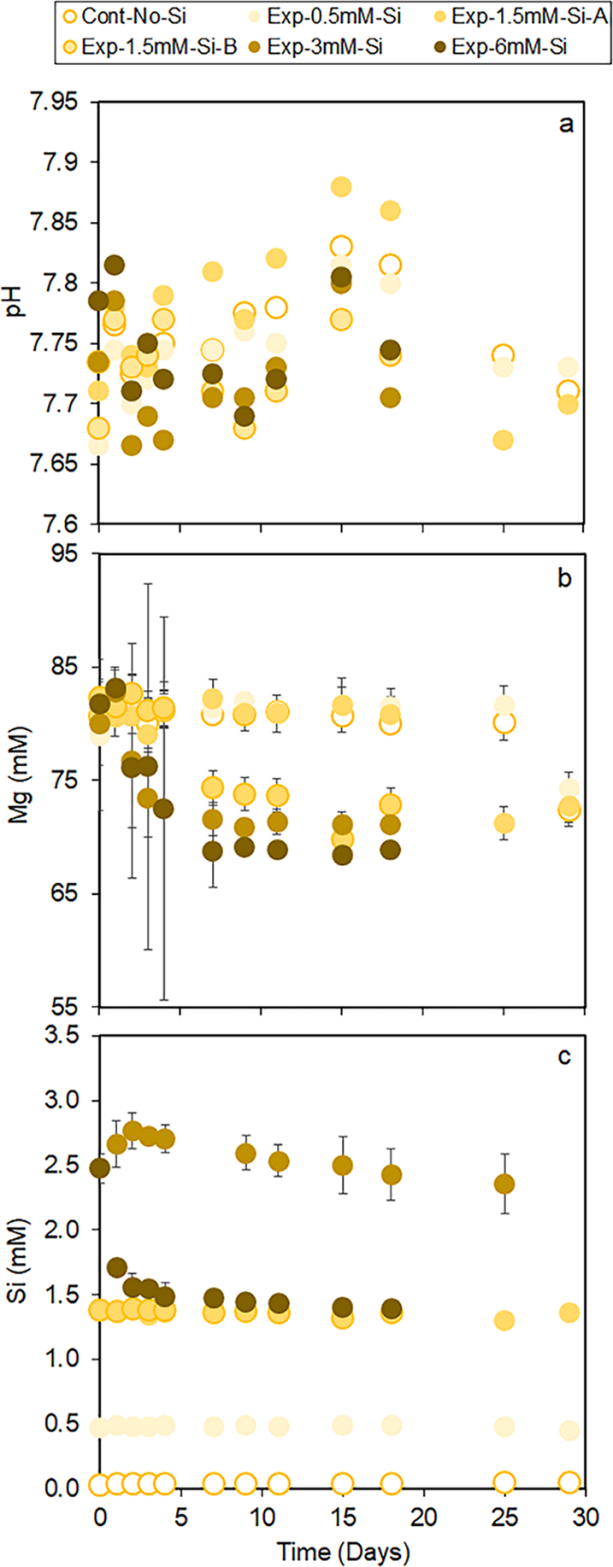
Temporal evolution of (a) pH and (b) Mg concentration and Si for all precipitation experiments. All uncertainty bars represent 2SD. 2SD on graph (a) represents analytical uncertainty derived from the repeatability of two independent experiments, whereas that on graphs (b) and (c) represents the analytical uncertainty for an individual measurement by ICP-OES or uncertainty derived from the repeatability of two independent experiments, whichever was larger. For plots where no uncertainty is visible, the uncertainty is smaller than the symbol. It is important to note that a low amount of Si (∼0.032 mM) was detected at time 0 days in the Cont-no-Si experiment (graph c). We confirmed that this Si originated from trace impurities present in the MgCl_2_·6H_2_O (Sigma-Aldrich, ACS grade) reagent used in this study. Nevertheless, this low Si concentration remained relatively stable throughout the duration of the Cont-no-Si experiment, indicating negligible Si released or adsorbed by the glass bottles during the experiments. The initial Si concentration in Exp-6 mM-Si was lower (∼2.5 mM) than the intended 6 mM and could indicate prior Si polymerization in this experiment. However, an independent control experiment conducted with 1.5 mM Na_2_SiO3·9H_2_O and 0.08 M MgCl_2_·6H_2_O (no NaHCO_3_ and NaCl) showed no change in Si concentration throughout the experiment, indicating the absence of Si polymerization at low initial Si concentrations (Figure S2).

By the fourth day of the experiment, visible precipitation was observed in both duplicates of Exp-3 mM-Si and Exp-6 mM-Si as well as in one of the duplicates of Exp-1.5 mM-Si-B. This was evident either as a white, cloudy suspension or for Exp-3 mM-Si-B as small precipitates at the air–water interface and attached to the glass walls of the reaction bottle. These five bottles remained the only ones to have precipitated until the 18th day, when the experiment was partially stopped. One of each duplicate from the experiments without precipitation was allowed to continue reacting. For these systems, precipitation was observed at 23 days (Exp-1.5 mM-Si-A), 25 days (Cont-no-Si-B), and 30 days (Exp-0.5 mM-Si-B). There is a considerable variation in the starting time of precipitate formation among the duplicates of Cont-No-Si, Exp-0.5 mM-Si, and Exp-1.5 mM-Si. We attribute this either to microscopic variations among duplicates that were not apparent at a macroscopic level or to the stochastic nature of nucleation in the experiments, which can lead to different initiation times due to random fluctuations. A summary of the time for precipitation and the weights of the solids extracted can be found in Table 1. Due to the presence of NaHCO_3_^–^ as a buffer, the initial pH values of all experiments were similar (ranging from 7.67 to 7.79, with slight variations likely due to variable Na_2_SiO_3_·9H_2_O concentrations in the experiments or due to measurement errors, Figure 1a). Notably, precipitation always coincided with a drop in the pH. This is consistent with a decrease in the Mg^2+^ concentrations in solution as well as a decrease in the Si concentrations in Exp-6 mM-Si (Figure 1b). It is important to note that in Exp-6 mM-Si, the initial Si concentration was lower (∼2.5 mM) than the intended 6 mM, and could indicate polymerization of Si prior to the experiment. However, no similar drop related to times of precipitation was observed in the lower concentration experiments. Instead, there is a gradual decrease in Exp-3 mM-Si and both duplicates show the same Si concentration trend in solution as Exp-1.5 mM-Si, even though a precipitate had already formed in the second duplicate (Exp-1.5 mM-Si-B) after 4 days. Based on the thermodynamic calculations, Mg-silicates (specifically, sepiolite (Mg_4_Si_6_O_15_(OH)_2_·6H_2_O)) should be supersaturated in all experiments that contained silica and, as such, would be expected to precipitate in all of the experiments. The yield of these phases should increase with increasing Si content in the growth solution as their saturation state also was observed to increase based on the simulations. In comparison, the Mg carbonates (magnesite, hydromagnesite, and lansfordite) are expected to be saturated at the temperature used in the experiments and a pH of 7.7 similar to that observed in the experiments.

**Figure 2:**
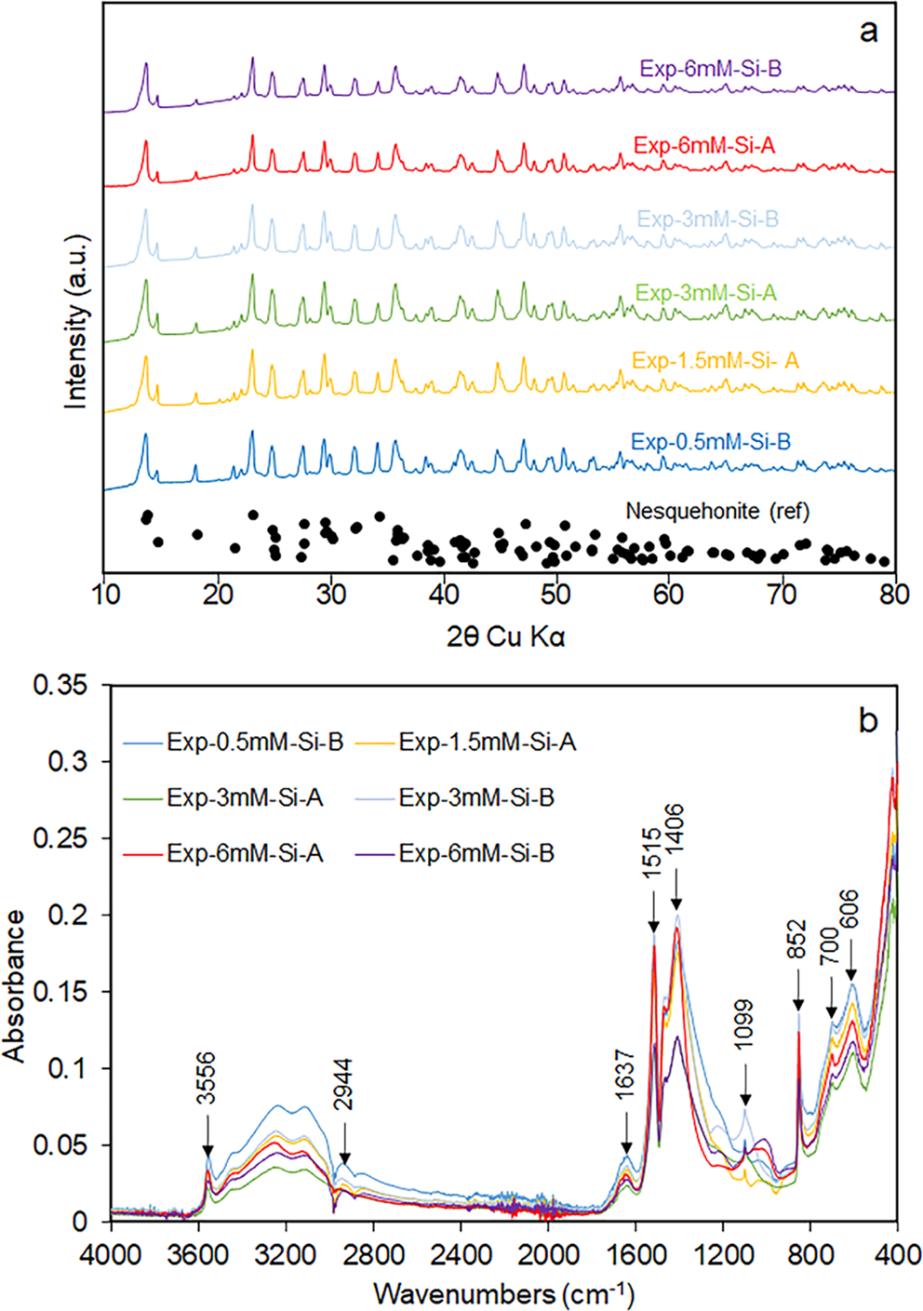
XRD peaks (a) and ATR-FTIR spectra (b) of the precipitated solids obtained from various experiments. Black circles in (a) indicate the position and height of the reference nesquehonite XRD peak obtained from the American Mineralogist Crystal Structure Database.

**Figure 3:**
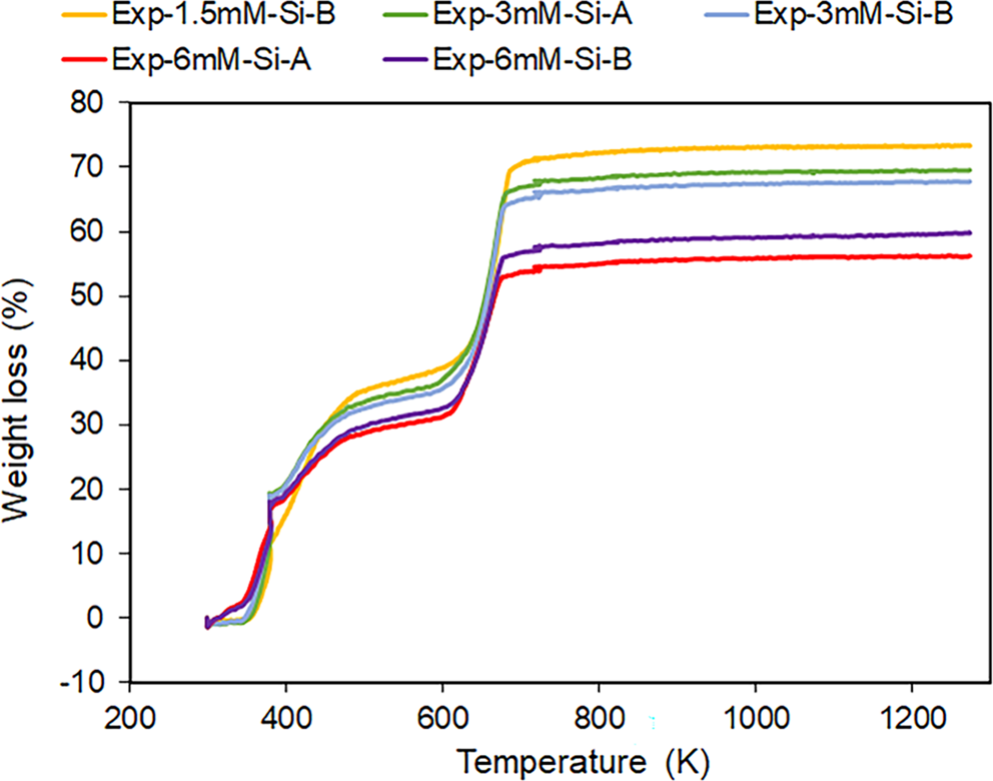
Thermogravimetric analysis (TGA) curves of the experiments with more than 1.5 mM silica in the growth solution.

**Figure 4:**
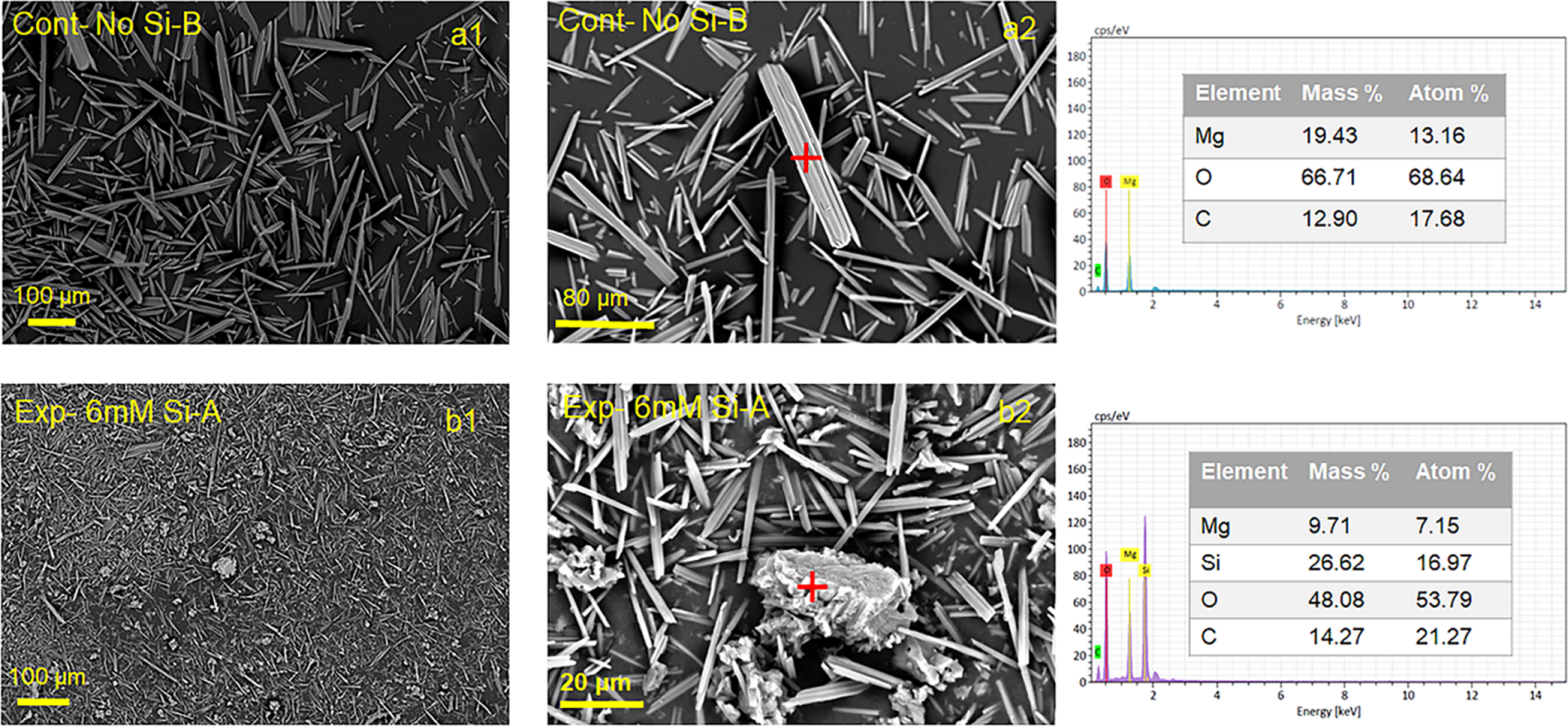
SEM image of precipitates obtained from Cont-No-Si-B (a1, a2) and Exp-6 mM-Si-A (b1, b2) experiments. EDS analyses of the precipitate with needlelike morphology in Cont-No-Si-B experiment in (a2), and the aggregate with irregular shape in (b2).

Despite the potential for the system to form Mg silicate phases such as sepiolite, a clear silica-related band (1000 cm^–1^ in Figure 2b) was only observed in the ATR-FTIR spectrum of the experiments with the highest concentration of silica in the growth solution. This band corresponds with that observed in the spectra of sepiolite related to Si–O stretching.^39^ No silicate minerals were observed in the XRD measurements from any of the experiments (Figure 3). All other precipitates formed fit well with the expected band positions found in the literature for nesquehonite^40,41^ and were confirmed by both XRD (Figure 2a) and Raman spectroscopy (Figure S3). This fits with the observations from Hänchen et al.^16^ who observed that only kinetically stable Mg-carbonate phases such as nesquehonite, rather than the thermodynamically most stable phase magnesite, form under similar conditions to our experiments. Only the experiments with over 1.5 mM silica in the solution produced enough material to also conduct TGA analysis. All three of these experiments showed a significant weight loss between 580 and 750 K (Figure 3), which can be attributed to the decarbonation of Mg carbonates.^42^ Again, a change in the overall weight loss in the TG curves for the experiments with the highest silica content supports the coprecipitation of a Mg silicate phase. Moreover, Exp-3 mM-Si showed a lower weight loss than expected for pure nesquehonite (70.9% based on the chemical formula MgCO_3_·3H_2_O)^43^ also indicating that coprecipitation of a noncarbonate material occurred in these experiments, which may be related to a very small silica-related band in the FTIR spectra of this material. SEM/EDS analysis confirmed the precipitation of needle-shaped nesquehonite crystals in all experiments and the coprecipitation of a Mg silicate phase (as irregularly shaped aggregates) in experiments with the highest silica content (Figure 4).

Incorporation of silica into the Mg-carbonate structure may also explain the observation that some Si was lost from solution. Experiments have shown that increasing sulfate concentrations in solution correlates with increasing full width at half-maximum (fwhm) in the XRD pattern of calcite, indicating that sulfate is incorporated into the calcite crystal structure.^44,45^ However, in our experiments, there was no change in the fwhm or the position of the nesquehonite XRD peaks with increasing silica concentrations in the original solution. Nor was there a change observed in the Raman peak positions and full width at half-maximum (fwhm) when nesquehonite was grown in the absence or presence of silica. Similarly, no change in the decarbonation behavior was observed in the TGA (Figure 3). There were differences in the dehydration temperatures below 400 K. However, weight loss in this region will also have a contribution from the silica phase observed in some of the ATR-FTIR spectra, which is probably hydrated; thus, a change in weight loss does not directly demonstrate a change in the nesquehonite phase. Based on the lack of change in the nesquehonite characteristics, we propose that the amounts of silica incorporated into the Mg-carbonate structure during the experiments were insignificant. Limited uptake of silica into the carbonate structure is also supported by the previous work of Guermech et al.,^46^ who precipitated nesquehonite and transformed it to hydromagnesite from and within a carbonated aqueous leach from serpentine mine wastes. Although the leach solutions contained up to 236 mg/L Si (3 wt % of ions in solution), only 0.7% of the nesquehonite and hydromagnesite chemical composition was Si based on total digestion of the precipitates.

### 3.2 Transformation Experiments

**Figure 5:**
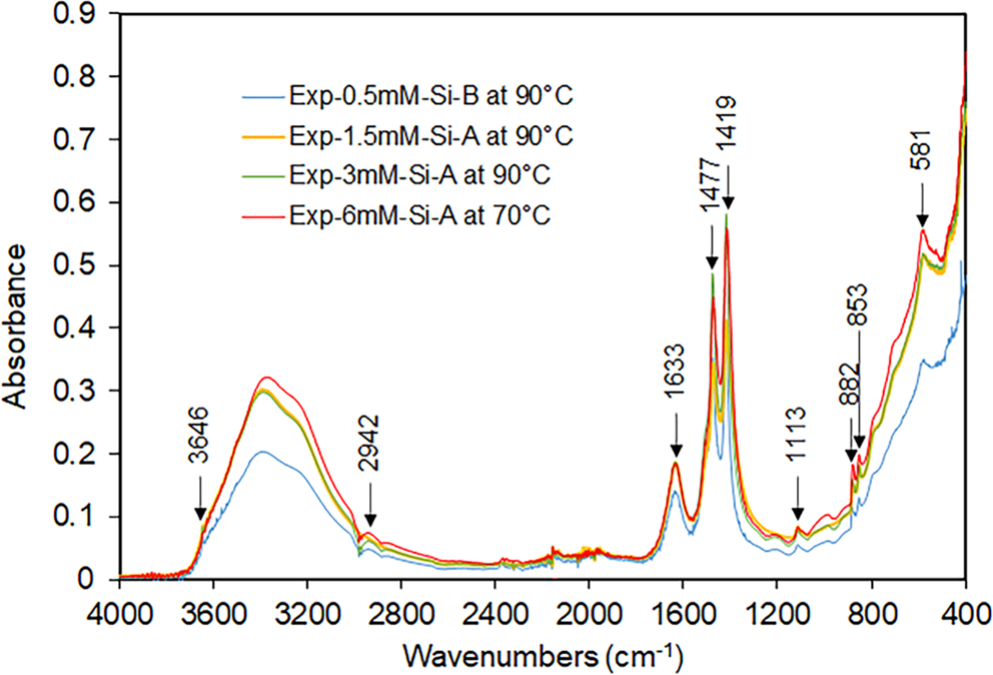
ATR-FTIR spectra of Exp-0.5 mM-Si-B, Exp-1.5 mM-Si-A, Exp-3 mM-Si-A, and Exp-6 mM-Si-A after being heated at 90 or 70 °C, respectively.

**Figure 6:**
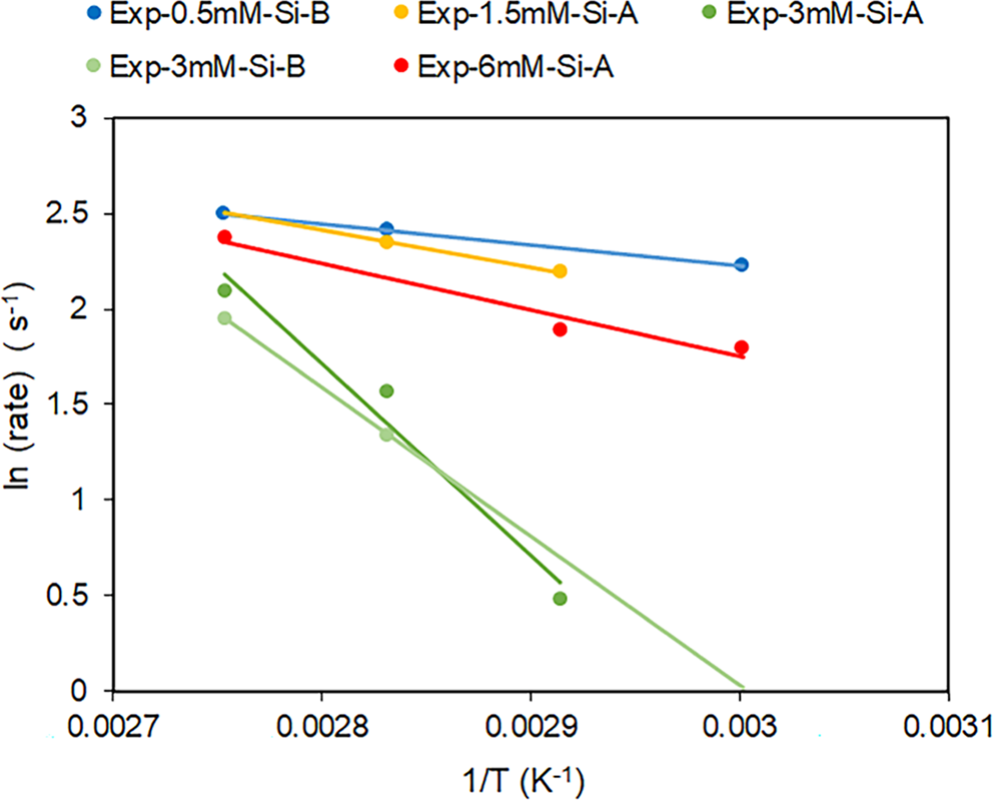
Arrhenius plot of the ln(rate) as a function of 1/*T*. The activation energy for each experiment is calculated from the slope of the line (Table S2).

Both dry and wet samples were heated at temperatures of up to 90 °C to test for changes in the stability of nesquehonite to transformation. Heating in air for over an hour showed no evidence for transformation of the nesquehonite produced in our experiments regardless of whether silica was present in the growth solution (Figure S4). This is consistent with the findings from Hopkinson et al.,^31^ who found that this transformation takes 8 days at 60 °C and 14 days at 52 °C under dry conditions. However, when water is added to the system, transformation did occur (Figure 5). This is evident from the changes in the antisymmetric stretching bands between 1400 and 1500 cm^–1^, a shift in the symmetric stretching mode to 1113 cm^–1^, and the presence of out-of-plane bending modes at 882 and 853 cm^–1^. These band positions are all consistent with the formation of hydromagnesite,^47^ as confirmed by Raman spectroscopy (Figure S5). Temperature and the presence of silica in the growth solution influenced the rate of transformation, which was measured based on the ratio between the intensity of the symmetric stretching mode for nesquehonite (1099 cm^–1^) and hydromagnesite (1113 cm^–1^), as these bands do not overlap in spectra with a mixture of the two materials. The rate was taken from the moment prior to the occurrence of measurable hydromagnesite in the spectrum and the plateau at which there was no more observable change in the ratio, typically because nesquehonite was no longer observable. The temporal evolution of the nesquehonite and hydromagnesite peak intensity is shown in Supporting Information, Figure S6. The rates are described in Supporting Information, Table S1 and plotted in an Arrhenius plot (Figure 6). As can be seen in Figure 6, higher temperatures resulted in a faster transformation of nesquehonite to hydromagnesite. In addition, nesquehonite grown in Exp-3 mM-Si took the longest time to transform at all temperatures tested, whereas nesquehonite formed in Exp-0.5 mM-Si transformed the fastest. However, nesquehonite grown in Exp-6 mM-Si does not fit this trend as it shows a rate that is closer to that of Exp-0.5 mM-Si and Exp-1.5 mM-Si. This can be explained by the change in the dissolved silica in the system through the precipitation of a Mg silicate phase. The dissolved silica concentration in Exp-6 mM-Si decreased on average until it was equivalent to that of Exp-1.5 mM-Si (Figure 1b). In contrast, Exp-3 mM-Si had a consistently higher silica concentration in the solution throughout the growth of the nesquehonite, indicating that this is more important than the initial concentration of silica for dictating the transformation behavior of nesquehonite.

### 3.3 Silica as an Agent to Promote Mg-Carbonate Precipitation and Stabilize Its Transformation

Our observations showed that the ability of nesquehonite to precipitate was altered in the presence of silica, which is consistent with previous observations where silica promoted the spontaneous precipitation of Ca carbonates^48^ and dolomites (Ca–Mg carbonates) in seawater-like solutions.^49^ Although it is clear that silica influences the processes occurring during nesquehonite precipitation and transformation, we do not have direct evidence of the mechanism by which silica changes this system. As our bulk analyses do not show any evidence that silica influences the atomic structure of nesquehonite, this implies that silica must influence the interfacial properties. For nesquehonite nucleation, two scenarios are possible depending on if the mineral forms via a classical or nonclassical nucleation pathway (see Zahn^50^ for a review of these theories). If nesquehonite is formed via ion-by-ion addition (classical nucleation) and nucleates homogeneously, then changes to the interfacial energy upon silica adsorption would be important in governing the critical radius of the particle needed to initiate precipitation, i.e., the transition state of the system. If silica continued to be adsorbed to the growing nesquehonite at specific surfaces, then we would expect to observe morphological changes. However, nesquehonite crystals showed no significant change during SEM imaging between the Cont-No-Si and Exp-6 mM-Si crystals (Figure 4), implying that, if present, silica does not show a high preference for specific surfaces. Alternatively, heterogeneous nucleation may also play a role in our system, as a Mg silicate phase formed in experiments with the highest silica concentrations. Hence, it is possible that colloids, clusters, or nanoparticles of silica may be increasingly present in our solutions as the silica concentration rises. In this case, interfacial energy-related constraints would be lowered if nucleation onto such a silica particle in solution occurred.

Silica is also known to stabilize prenucleation clusters of Ca carbonates^51^ that are responsible for the formation of critical nuclei via nonclassical nucleation pathways. Particularly at pHs lower than 9.3, which is the region of the experiments described here, silica stabilized amorphous prenucleation clusters of Ca-carbonate that agglomerated to form larger and eventually crystalline materials. However, if heterogeneous nucleation or cluster agglomeration would occur, silica should be lost from the solution upon nesquehonite crystal formation and there is no evidence for this in the silica solution concentrations in the 1.5 or 0.5 mM silica experiments, where there is also no evidence for the formation of a separate silica-bearing phase. Therefore, we can only assume that the presence of silica can disrupt the water-Mg surface complex and enhance dehydration at the surface, enhancing precipitation, as proposed by Fang and Xu.^49^

Clearly, the effect of silica also plays a role in the transformation of nesquehonite to hydromagnesite. This transformation is associated with a concomitant change in the particle morphology (Figure S7) and was significantly faster when conducted in water, consistent with the solvent-mediated dissolution-reprecipitation pathway proposed previously.^20,52^ In transformations governed by dissolution and precipitation reactions, changing the rate-limiting step will alter the overall rate of the process. If a system is dissolution-controlled, then typically a pseudomorph will form of the original crystal.^53^ However, if the rates of dissolution and reprecipitation are similar or the system is precipitation-controlled, then the two reactions will become spatially uncoupled in an unconfined system. Thus, the formation of a new shape implies that this reaction is precipitation-controlled and that silica adsorption affects hydromagnesite in a similar manner to nesquehonite.

### 3.4 Implications for Carbon Sequestration Methods That Rely on the Formation of Hydrated Mg Carbonates

Yields of nesquehonite, Mg silicate phase, and CO_2_ stored in each experiment can be approximated by calculating the amount of carbonate mineral within a given weight using the TGA data (Table 2 and Supporting Information). Even though the relative CO_2_ amount in Exp-6 mM-Si is the lowest measured (only 23.3–25.3%), this value combined with the total weight of the sample obtained, means that the experiments with the highest silica content in the growth solution yielded the highest amount of nesquehonite, thus being the most effective at removing the CO_2_ (97.5 mg, Table 2). However, Harrison et al.^20^ observed that CO_2_ is released upon transformation of nesquehonite to hydromagnesite. Thus, additional stabilization of the Mg-carbonate product is also desirable. Formation of significant amounts of a Mg silicate phase in Exp-6 mM-Si means that this effect is limited. In fact, Exp-3 mM-Si are the most effective at sequestering CO_2_, as they can produce a similar overall yield to that of the experiment with double the silica content (cf. Exp-3 mM-Si-A and Exp-6 mM-Si-A in Table 2) and twice as much yield as that of the experiments with half the silica content (Exp-1.5 mM-Si-B) during the experiment duration. This system showed the most resistance to thermal transformation through a dissolution-reprecipitation pathway with an activation energy of 64.912 kJ/mol (Table S2). Thus, would be expected to result in the most stable Mg-carbonate material. Overall, our findings indicate that the precipitation of silica-assisted Mg carbonates could be a feasible option for carbon sequestration. Clearly, the relative rates of Mg-carbonate and Mg silicate are important in these processes and more work is needed to assess whether this relationship will change at lower concentrations of carbonate, thus a lower thermodynamic driving force for carbonate formation.

**Table 2:** Quantities of Nesquehonite, Mg Silicate Phase, and Sequestered CO_2_ in Milligrams and Weight Percentage in Various Experiments Based on TG Analyses^a^

experiment	wt nesquehonite (mg)^b^	wt % nesquehonite	wt Mg silicate (mg)^b^	wt % Mg silicate	wt CO_2_ sequestered (mg)^c^	wt % CO_2_ sequestered
Exp-1.5 mM-Si-B	138	100	0	0	43.9	31.8
Exp-3 mM-Si-A	263	97.4	7	2.57	83.6	30.98
Exp-3 mM-Si-B	206	94.7	12	5.53	66	30.04
Exp-6 mM-Si-A	285	73.1	105	26.9	90.5	23.3
Exp-6 mM-Si-B	307	79.6	78.5	20.4	97.5	25.3

a Details of the calculation are provided in the Supporting Information.

b Derived from the difference between pure nesquehonite based on the formula weight of nesquehonite and the total weight loss based on TGA data.

c Derived from the amount of CO_2_ that pure nesquehonite can store and the amount of nesquehonite in each experiment.

## Supporting Information Available

Calculations for the weight (%) of nesquehonite and the corresponding amount of CO_2_ stored using the TGA data; FTIR spectrum of water; independent control experiment with only 1.5 mM Na_2_SiO_3_·9H_2_O and 0.08 M MgCl_2_·6H_2_O; Raman spectra of Cont-No-Si-A, Exp-0.5 mM-Si-A, Exp-1.5 mM-Si-B, and Exp-6 mM-Si-A at room temperature; FTIR spectra of dry precipitated samples from Exp-0.5 mM-Si-B and Exp-6 mM-Si-A after being heated at 90 °C for 1 h; temporal evolution of nesquehonite (Ne) and hydromagnesite (HM) peak’s intensity for wet samples from Exp-1.5 mM-Si-A at 90 °C, Exp-3 mM-Si-A at 70 °C, and Exp-6 mM-Si-A at 70 °C; Raman spectra of Exp-1.5 mM-Si-A after being heated at 70 °C, Exp-3 mM-Si-A after being heated at 90 °C, and Exp-6 mM-Si-B after being heated at 70 °C; microscopic images of the precipitates from Exp-3 mM-Si-A at 90 °C and Exp-1.5 mM-Si-A at 70 °C showing typical hydromagnesite morphology; rates of change from nesquehonite to hydromagnesite for Exp-0.5 mM-Si-B, Exp-1.5 mM-Si-A, Exp-3 mM-Si-A, Exp-3 mM-Si-B, and Exp-6 mM-Si-A at 60, 70, 80, and 90 °C; activation energies (kJ/mol) calculated for Experiments Exp-0.5 mM-Si-B, 2A, Exp-3 mM-Si-A, Exp-3 mM-Si-B, and Exp-6 mM-Si-A; and thermal decomposition data from TG analyses (PDF)
