# Supplementary material for: Enhanced Nesquehonite Formation and Stability in the Presence of Dissolved Silica
Source: Environ Sci Technol. 2023 Dec 27;58(1):362–70. doi: 10.1021/acs.est.3c06939 (PMC10785746; doi:10.1021/acs.est.3c06939)
Supplement: Supplementary file 1 — es3c06939_si_001.pdf [file es3c06939_si_001.pdf]

Supporting information

Enhanced nesquehonite formation and stability in the presence of dissolved silica

Rasesh Pokharel[1,2,†,], Iasmina C. Popa[1,†], Yannick de Kok[1], Helen E. King[1]*

[1]Department of Earth Sciences, Utrecht University, Princetonlaan 8a, 3584CB, Utrecht, The Netherlands.

[2]Copernicus Institute of Sustainable Development, Utrecht University, Princetonlaan 8a, 3584CB, Utrecht, The Netherlands

*Corresponding author: Tel: +31 30 253 1409 and email: r.pokharel@uu.nl

Number of pages: 10

Number of figures: 7

Number of tables: 3

Calculations for the weight (%) of nesquehonite and the corresponding amount of CO_2 stored using the TGA data

Molar mass:

Nesquehonite: $MgCO_3 \cdot 3H_2O$ = 138.36g/mol

Sepiolite: $Mg_4Si_6O_{15}(OH)_2 \cdot 6H_2O$ = 647.82g/mol

Hydromagnesite: $(MgCO_3)_4Mg(OH)_2 \cdot 4H_2O$ = 467.631g/mol

H_2O: 18.0147g/mol

CO_2: 44.009g/mol

Based on the molar mass, the amount of H_2O and CO_2 in nesquehonite, sepiolite and hydromagnesite are as follows:

**Mineral**	**H_2O** %	**CO_2** %
Nesquehonite	39.061	31.8
Sepiolite	16.685	-
Hydromagnesite	15.409	37.644

The amounts (%) of nesquehonite (x) and sepiolite-like (y) phases in Exp-3mM-Si and Exp-6mM-Si were calculated using the weight loss from the TGA analysis (Table S3). The amounts of nesquehonite and sepiolite-like phases present need to sum up to 100%, so $x + y = 1$. Thus:

Exp-3mM-Si-A: $70.861x + 16.685y = 69.4818$

$x = 97.43$ % and $y = 2.57$ %

Exp-3mM-Si-B: $70.861x + 16.685y = 67.874$

$x = 94.47$ % and $y = 5.53$ %

Exp-6mM-Si-A: $70.861x + 16.685y = 56.3099$

$x = 73.13$ % and $y = 26.87$ %

Exp-6mM-Si-B: 70.861x + 16.685y = 59.8232

x = 79.612 % and y = 20.388 %

Therefore, the amount of CO_2 that each Experiment could store was calculated below, using the amount of CO_2 that pure nesquehonite can store and the amount of nesquehonite in each experiment (which was calculated above, the x values):

CO_2 stored in Exp-3mM-Si-A: 97.43 × 0.318 = 30.982 %

CO_2 stored in Exp-3mM-Si-B: 94.47 × 0.318 = 30.041 %

CO_2 stored in Exp-6mM-Si-A: 73.13 × 0.318 = 23.255 %

CO_2 stored in Exp-6mM-Si-B: 79.612 × 0.318 = 25.3166 %

Moreover, the weight of nesquehonite in each sample (in grams) can be calculated, knowing the total weight, and the amount of nesquehonite (%):

Weight of nesquehonite in Exp-3mM-Si-A: 97.43% × 0.27 = 0.263 g

Weight of nesquehonite in Exp-3mM-Si-B: 94.47% × 0.218 = 0.206 g

Weight of nesquehonite in Exp-6mM-Si-A: 73.13% × 0.389 = 0.2845 g

Weight of nesquehonite in Exp-6mM-Si-B: 79.612% × 0.385 = 0.3065 g

The weight of the Mg-silicate phase (in grams) can be obtained by subtracting the above-calculated weights of nesquehonite in each sample from the total weight of each sample.

Then, the weight of CO_2 that each sample can store (in grams) can be calculated, knowing the weight of nesquehonite and the amount of CO_2 that pure nesquehonite can store (i.e., 0.318):

CO_2 stored in Exp-3mM-Si-A: 0.263 g x 0.318 = 0.0836 g

CO_2 stored in Exp-3mM-Si-B: 0.206 g x 0.318 = 0.066 g

CO_2 stored in Exp-6mM-Si-A: 0.2845 g x 0.318 = 0.0905 g

CO_2 stored in Exp-6mM-Si-B**:** 0.3065 g x 0.318 = 0.0975 g

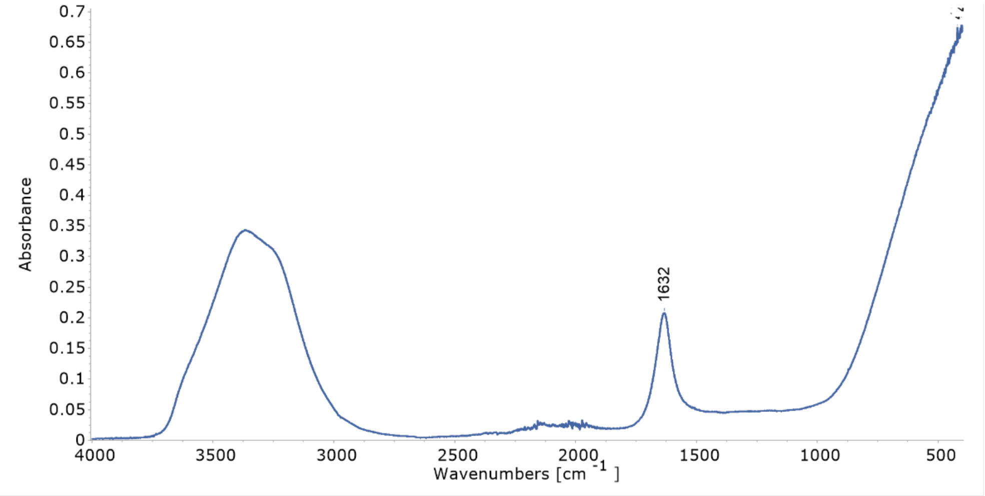

Figure S1: FTIR Spectrum of Water, showing the stretching mode of OH units in the region between 3000-3600 cm^{-1}, and the OH bending mode in the region of 1600 cm^{-1}.

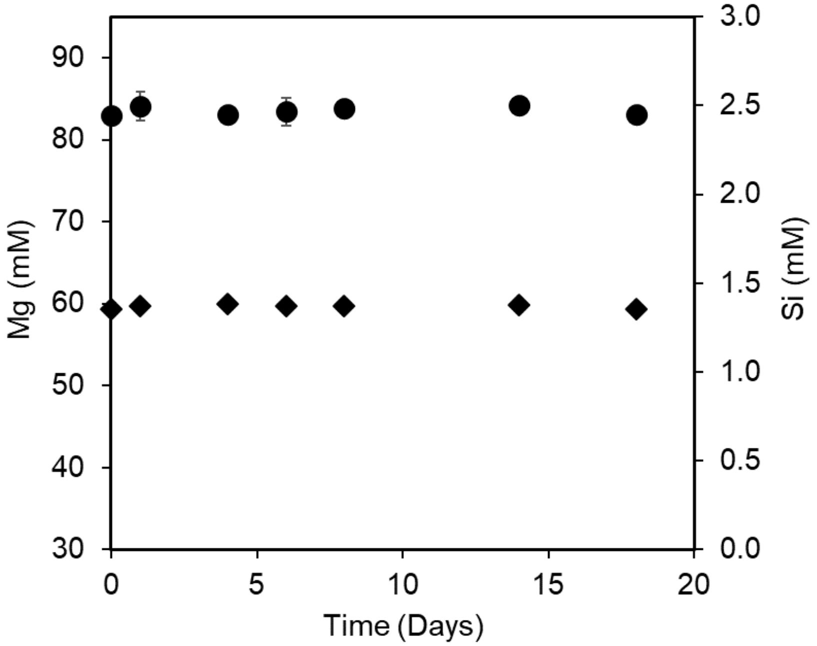

Figure S2: Temporal evolution of Mg (black circles on the primary Y-axis) and Si (black squares on the secondary Y-axis) in an independent control experiment conducted with 0.08 M $MgCl_2 \cdot 6H_2O$ and 1.5 mM $Na_2SiO_3 \cdot 9H_2O$ (without NaCl and $NaHCO_3$). The error bar represents analytical uncertainty (2SD) derived from the repeatability of two independent experiments. Throughout the experiment, both Si and Mg concentrations remained constant.

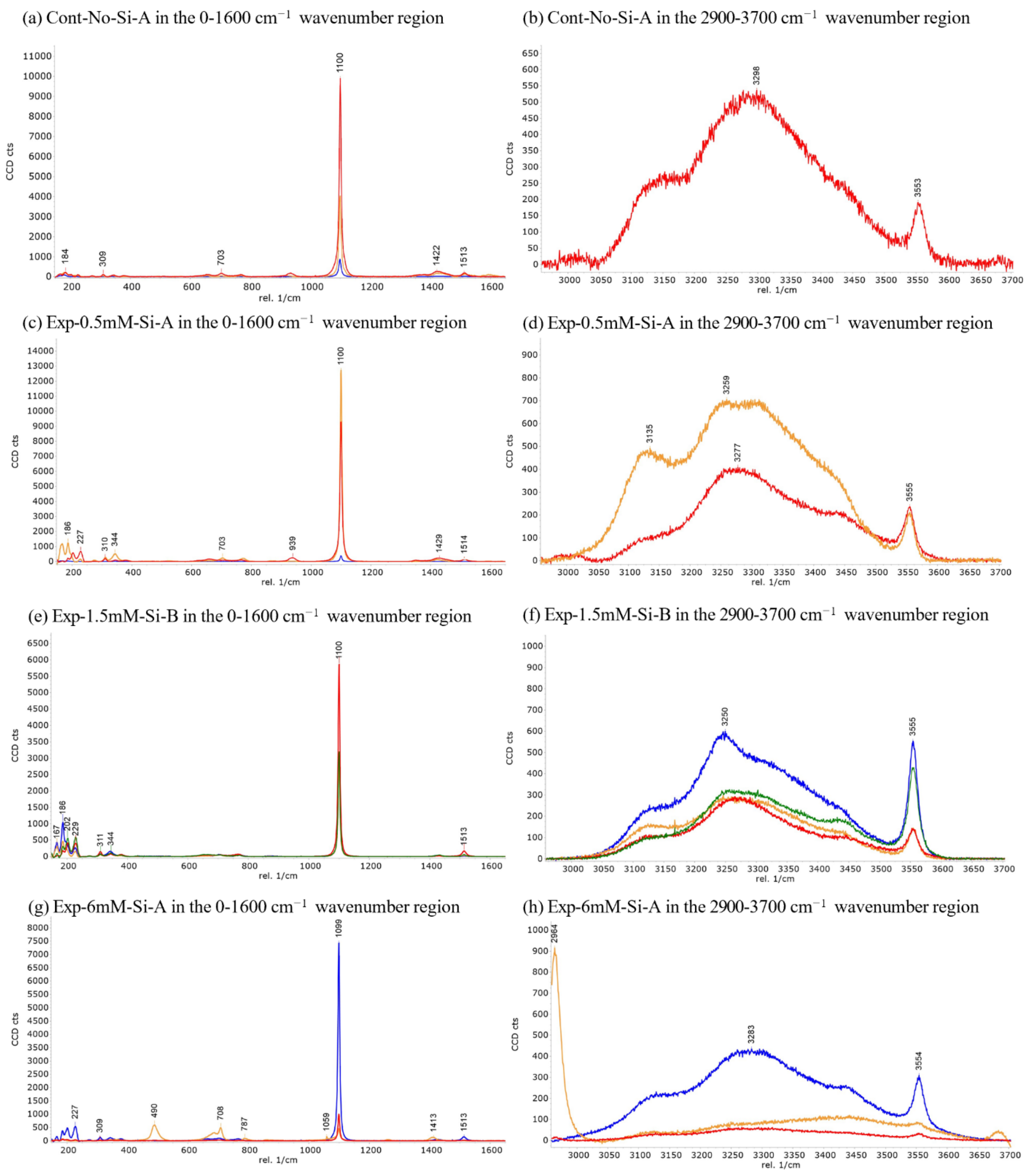

Figure S3: Raman spectra of Cont-No-Si-A (panel a and b), Exp-0.5mM-Si-A (panel c and d), Exp-

1.5mM-Si-B (panel e and f) and Exp-6mM-Si-A (panel g and h) at room temperature. Each colour represents different measurements.

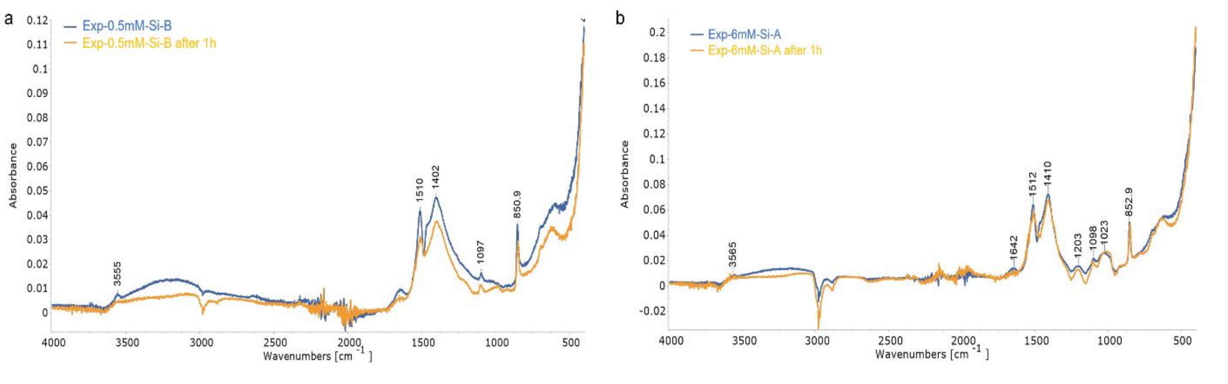

Figure S4: FTIR spectra of dry precipitated samples from Exp-0.5mM-Si-B (panel a) and Exp-6mM-Si-A (panel b) after being heated at 90°C for 1h.

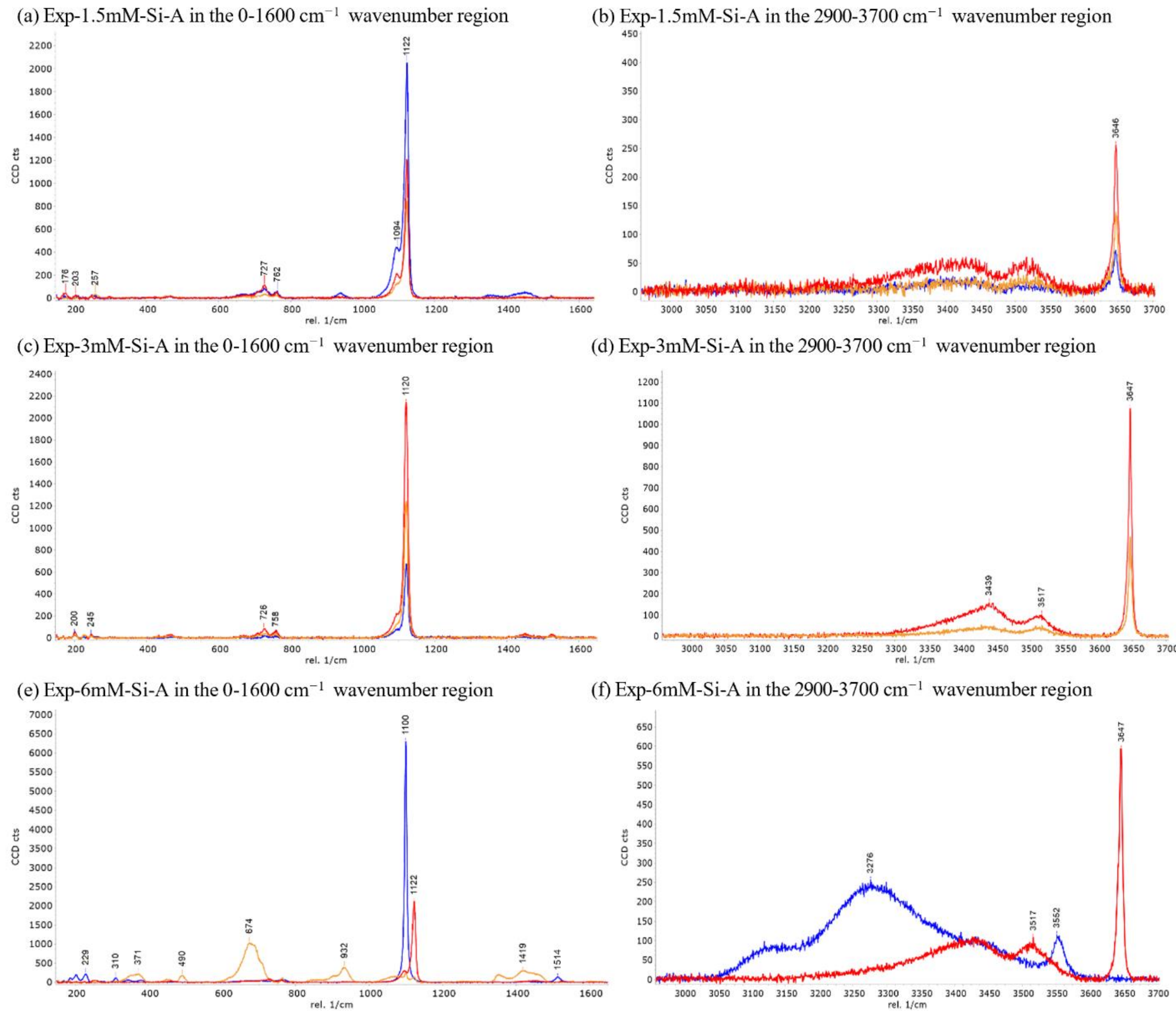

Figure S5: Raman spectra of Exp-1.5mM-Si-A (panel a and b) after being heated at 70°C; Exp-3mM-Si-A (panel c and d) after being heated at 90°C; and Exp-6mM-Si-B (panel e and f) after being heated at 70°C. Each colour represents a different measurement.

Note: The Raman spectra of Experiment 6mM show that this sample contains three different phases: nesquehonite (blue spectra), hydromagnesite (red spectra), and the silica phase (yellow spectra). However, the IR spectrum of Experiment 4A at 70°C does not show any nesquehonite bands, its presence being picked up only by the Raman spectroscopy. This implies that, if still present, the traces of nesquehonite are below the detection limit of the IR spectroscopy. Therefore, the presence of nesquehonite in the Raman spectrum of Experiment 4A does not affect the transformation rates presented above, because the rates have been calculated based on the IR spectrum, which shows that the sample is fully transformed into hydromagnesite. Moreover, the silica phase is still present in the hydromagnesite in Experiment 4A.

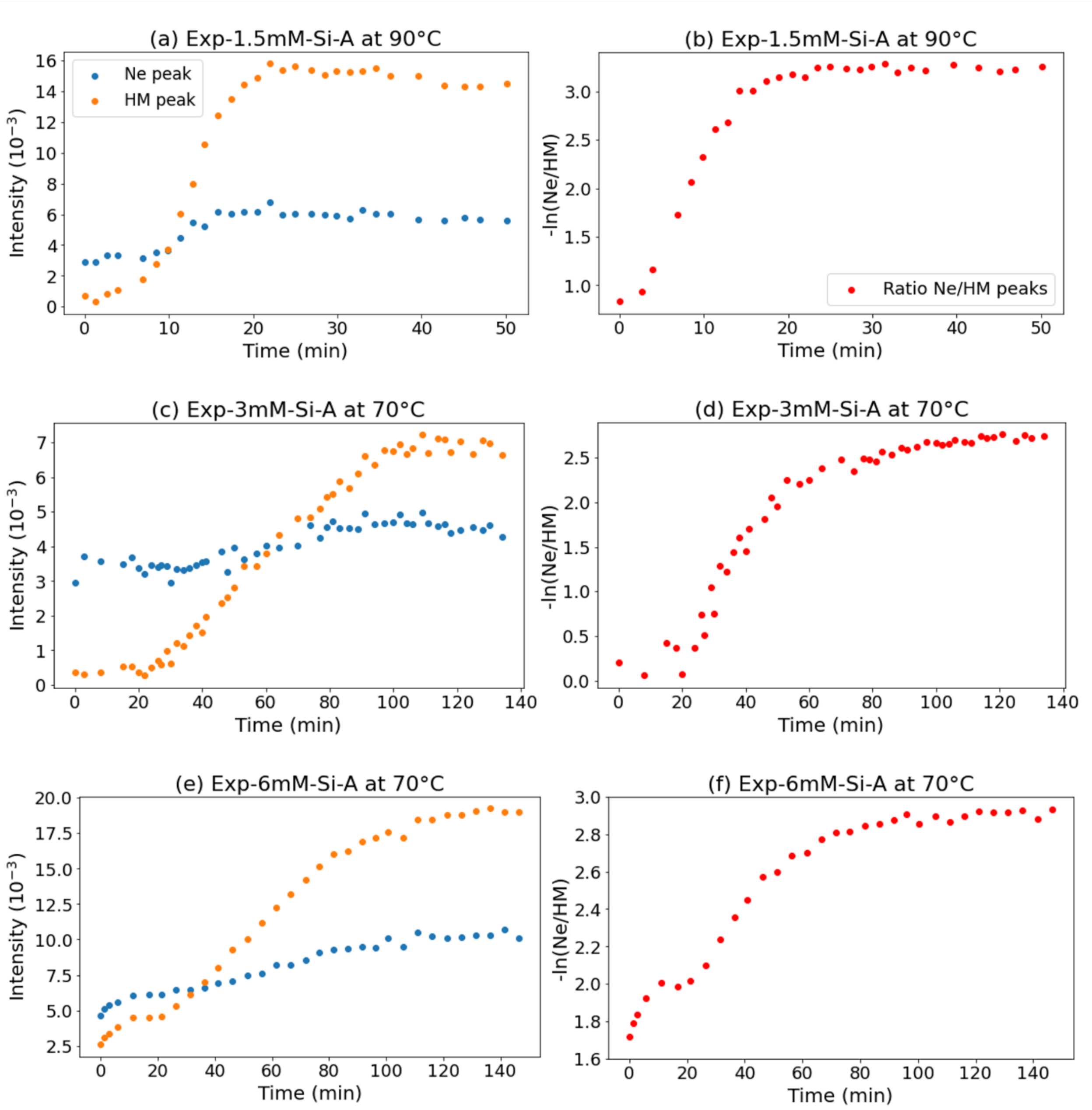

Figure S6: Temporal evolution of Nesquehonite (Ne) and Hydromagnesite (HM) peak's intensity for wet samples from Exp-1.5mM-Si-A at 90°C, Exp-3mM-Si-A at 70°C and Exp-6mM-Si-A at 70°C.

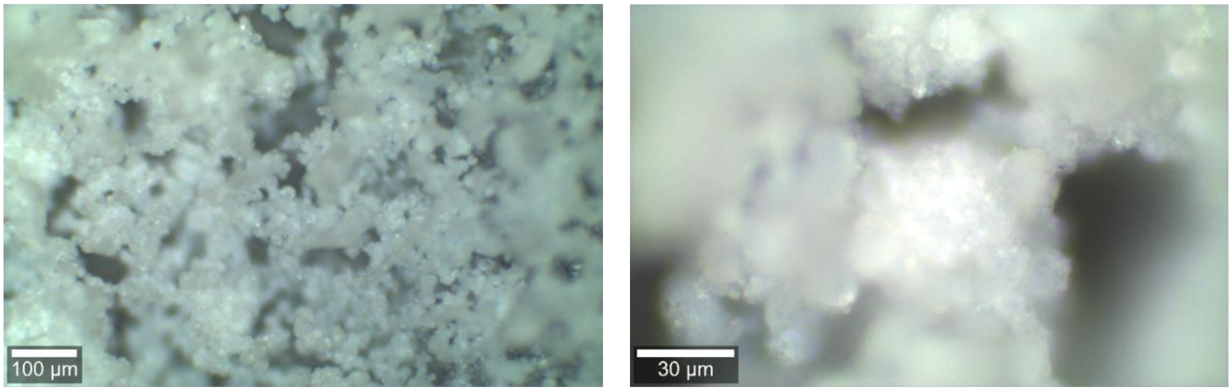

Figure S7: Microscopic images of the precipitates from Exp-3mM-Si-A at 90°C (left) and Exp-1.5mM-Si-A at 70°C (right) showing typical hydromagnesite morphology.

Table S1: The rates of change from nesquehonite to hydromagnesite for Exp-0.5mM-Si-B, Exp-1.5mM-Si-A, Exp-3mM-Si-A, Exp-3mM-Si-B and Exp-6mM-Si-A at 60°C, 70°C, 80°C and 90°C.

Experiment	60°C	70°C	80°C	90°C
Exp-0.5mM-Si-B	2.22	Not done	2.41	2.49
Exp-1.5mM-Si-A	Not done	2.19	2.35	-**
Exp-3mM-Si-A	Not done	0.48	1.57	2.09
Exp-3mM-Si-B	-*	Not done	1.34	1.95
Exp-6mM-Si-A	1.80	1.89	Not done	2.37

Note: According to Raman measurements, *Exp-3mM-Si-B was still nesquehonite after being heated at 60°C for 4h using the ATR-FTIR instrument. Therefore, it did not transform into hydromagnesite within this time and therefore its rate was not considered when making the Arrhenius plot. This is why Exp-3mM-Si-B in the Arrhenius plot (Figure 6) has only two rates, at 90°C and 80°C. **Exp-1.5mM-Si-A was heated at 90°C for 1h 30 min in which time it had transformed into hydromagnesite according to the FTIR spectra (Figure 5). However, its rate showed significant deviation from all the others, being around 0.9 and could not be repeated thus we have not included this rate in the calculations as there was an unknown experimental error associated with this experiment.

Table S2: Activation energies (kJ/mol) calculated for Experiments Exp-0.5mM-Si-B, Exp-1.5mM-Si-A, Exp-3mM-Si-A, Exp-3mM-Si-B and Exp-6mM-Si-A, based on the slope of the Arrhenius plot shown in Figure 6 in the publication itself.

Experiment	Activation Energy (kJ/mol)
Exp-0.5mM-Si-B	9.09
Exp-1.5mM-Si-A	16.12
Exp-3mM-Si-A	83.69
Exp-3mM-Si-B	64.91
Exp-6mM-Si-A	20.01

Table S3: Thermal decomposition data from TG analyses

Experiment	Total weight loss (%)	Weight loss in the dehydration stage below (580 K) (%)	Weight loss in the decarbonation stage below (580 - 750 K) (%)	Weight loss in the last stage below (750 – 1273 K) (%)
Exp-1.5mM-Si-B	73.32	38.07	33.53	1.7
Exp-3mM-Si-A	69.48	35.88	32	1.6
Exp-3mM-Si-B	67.87	34.88	31.3	1.7
Exp-6mM-Si-A	56.31	30.86	23.8	1.64
Exp-6mM-Si-B	59.82	32.08	25.7	2.05
